# Do pregnancy rates differ with intra-uterine insemination when different combinations of semen analysis parameters are abnormal?

**DOI:** 10.4274/jtgga.2017.0082

**Published:** 2018-06-04

**Authors:** Anita Kuriya, Chioma Agbo, Michael H. Dahan

**Affiliations:** 1 Division of Reproductive Endocrinology and Infertility, Department of Obstetrics and Gynecology, McGill University Health Center, Montreal, Canada; 2 Department of Emergency Medicine, Stanford University School of Medicine, California, USA

**Keywords:** Artificial insemination, pregnancy rate, semen analysis

## Abstract

**Objective:**

To evaluate the relationship of one or a combination of semen analysis parameter results on insemination outcomes.

**Material and Methods:**

A retrospective analysis was performed to evaluate the effect on pregnancy rates in relation to one or more abnormal semen analysis parameters based on the 2010 World Health Organization semen analysis guidelines.

**Results:**

Nine hundred eighty-one couples underwent 2231 intrauterine insemination cycles at the Stanford Fertility and Reproductive Medicine Center. In our study, the pregnancy rates ranged from 11-25% when an individual or combined semen analysis parameters were analyzed. Similar pregnancy rates were found when one, two, and in most cases three parameters were abnormal. When a single parameter was abnormal among volume, concentration, and motility, pregnancy rates were mainly unaffected. There was the exception of total sperm count where pregnancy rates were diminished when counts were below 39 million (p=0.04).

**Conclusion:**

Clearly, total sperm in the specimen and not the concentration of sperm per milliliter was the critical factor for predicting pregnancy. Therefore, a reorganization of semen analysis reports should be done emphasizing the total amount of sperm present and de-emphasizing concentration of sperm.

## Introduction

Infertility is the failure to conceive following twelve months of unprotected intercourse (1). Studies suggest that infertility affects 10 to 15% of the reproductive population (1). Male factor infertility is responsible for up to 50% of infertility cases (1). Male factor infertility is diagnosed primarily based on the results of at least two semen analyses performed 90 days apart. A semen analysis consists of a wide range of parameters including: volume, sperm concentration, progressive motility, and morphology. The total motile sperm count (TMSC) is calculated by multiplying the total sperm in the specimen by the percentage of motile sperm and is felt to be an essential predictor of intrauterine insemination (IUI) success (2).

When faced with severe male factor infertility, although there exists a lack of randomized control trials, the consensus is to offer in vitro fertilization (IVF) with intracytoplasmic sperm injection (ICSI) (3). Others argue that IVF should not be considered for routine use (3) and question its cost-effectiveness for most cases of male factor infertility (3). In these cases, it is argued that IUI should be the first-line treatment instead (3,4). 

Most male partners of couples presenting for infertility will have one or more abnormal parameters in their semen analysis. Many have studied the effect of single parameters in relation to pregnancy and fertilization outcomes (5,6,7,8), or combining some of these parameters into the TMSC (9,10,11,12). However, there exists a lack of literature on whether a combination of parameters or any specific parameter (except for TMSC) would allow for lower pregnancy rates with IUI. Therefore, the objective of this study was to evaluate the effect on pregnancy rates with one or more abnormal semen analysis parameters based on the 2010 World Health Organization (WHO) semen analysis guidelines. 

## Material and Methods

A retrospective analysis was performed on 2.5 years of data collected at an American University. A total of 981 couples underwent 2231 IUI cycles. The original database contained information regarding evaluation of semen quality on the day of insemination. Subjects at the clinic are 40% Caucasian, 7% African American, 33% Asian, and 20% Hispanic. The biochemical pregnancy rate was 14%, the clinical pregnancy rate was 82%, and the ectopic pregnancy rate was 4%. 

Semen quality was classified on the day of insemination based on the 2010 WHO semen criteria. Criteria used were 1.5 mL, 15 million/mL, minimum count per specimen 39 million, and forward motility 32%. If Kruger strict morphology was less than 4% in two samples, the patients were treated with IVF and ICSI. These patients were not included in this study. It should also be noted that strict morphology is not traditionally calculated on each specimen being used for insemination because preparation would kill some of the sample required for use, possibly affecting pregnancy rates. It should also be noted that total motility was not calculated by the computer semen analyzer and was therefore unavailable for comparison. The 2010 WHO criteria also list a minimum total motility of 40% in the specimen as criteria of normality. However, forward motility of 32% was used because this and not total percent motility was available in the processing report. The specimens were compared based on the presence of all criteria being normal or one or more being abnormal.

**The evaluation and examination of patients:** The couples enrolled in this study had at least one year of either primary or secondary infertility with their current partner. All couples underwent a comprehensive evaluation including medical history and physical examination, documentation of ovulation or an assessment for the lack thereof, as well as a semen analysis using Kruger strict morphology. All patients had at least one patent fallopian tube on either hysterosalpingogram or laparoscopy with chromopertubation. Ovulation was evaluated with a luteal phase progesterone >3 ng/mL, basal body temperature charts, urinary luteinizing hormone (LH) kits with regular cycles every 21 to 35 days, or regular periods every 21 to 35 days with a clear history of premenstrual molimina. All women had serum prolactin and thyroid-stimulating hormone levels in the normal range of the assay used before starting treatment. Women were included if they were anovulatory with inducible ovulation, if they had a serum follicle-stimulating hormone levels <12 IU/L on basal and clomiphene citrate challenge testing (if performed), a baseline follicle count of greater than 8 on endovaginal ultrasonography or stage 1-2 endometriosis on laparoscopy with at least one patent and undamaged fallopian tube. All women were evaluated with hysterosalpingography or hysteroscopy, and any intra-cavitary pathology including polyps, fibroids, and synechiae were corrected before initiating treatment. Any patients with four or more myometrial fibroids of 1 cm or greater in diameter, or one leiomyoma of 5 cm or greater in the uterine muscle, underwent surgical resection and appropriate recovery before initiating the insemination cycle.

Couples did not have, women with bilaterally blocked fallopian tubes, decreased ovarian reserve, stage 3 or 4 endometriosis, recurrent pregnancy loss (2 or more miscarriages), two previous ectopic pregnancies or anovulation and folliculogenesis was not successfully induced. Donor frozen IUI semen results were excluded because only post-processing parameters were available for these samples, and the donor was unlikely to be infertile. Only partners’ fresh sperm specimens were included in the analyses.

Seven percent of patients were treated with natural cycle IUI, 54% were treated with clomiphene IUI, 3% were treated with letrozole IUI, and 36% received gonadotropin IUI. Gonadotropin injections were performed daily starting on cycle day 2 or 3 and titrated to develop 2 to 3 mature follicles in patients aged under 40 years, and 2 to 5 follicles in women aged over 40 years. Clomiphene citrate (50 or 100 mg daily) and letrozole (5 mg daily) were administered orally for five days starting on cycle day 2 to 4. Serial sonography was performed to monitor folliculogenesis as per standard protocols.

**Semen collection, analysis, and processing:** Individuals were asked to refrain from ejaculation for two to four days before the collection of the specimen. Specimens were produced with masturbation, either in a collection room at the fertility clinic or at the patient’s home. To be collected at home, the sample had to be delivered within thirty minutes of production while being kept warm (i.e., placement of the receptacle in an axilla). 

Freshly ejaculated sperm was allowed to liquefy before semen analysis. Liquefied semen was thoroughly mixed before an aliquot was placed on a standard count slide (Leja Products BV, Nieuw-Vennep, the Netherlands) for the pre-processing analysis. The slide was placed on a 37 °C stage of an IVOS computer-assisted semen analyzer (Hamilton Thorn Biosciences, Beverly, MA). At least three random fields were evaluated for each analysis. Intra and inter-assay coefficients of variation of the parameters were less than 10% in all cases, pre- and post-processing.

Following the initial semen analysis, the sample was processed by first placing up to 4 mL of raw semen on a differential density gradient column consisting of 1 mL of 40% PureSperm and 1 mL of 80% PureSperm (Nidacon, Molndol, Sweden). The gradient was centrifuged for 20 minutes at 350 × g, and subsequently, the 40% layer and the seminal plasma fraction were removed from the test tube, leaving the 80% layer undisturbed. Approximately 6-8 mL of sperm-washing medium and 5% HAS (Cooper-Sage, Trumbull, CT) was added to the 80% layer and centrifuged for 10 minutes at 550 × g. The sperm pellet was then reconstituted to approximately 0.5 mL. The analysis of an aliquot of the processed sample was performed as previously described using the IVOS computer-assisted semen analyzer.

IUI and beta-human chorionic gonadotropin (β-hCG) assay: IUI was performed approximately 24-hours (+/- 3 hours) after detection of a spontaneous urinary LH surge, or 36-hours (+/- 1 hours) after 10.000 IU β-hCG injection (Pregnyl, Merck, West Orange, NJ), (Novarel, Ferring Pharmaceuticals, Inc., Tarrytown, NY) or 250 mcg Ovidrel injection, (Merck-Serono Laboratories, Rockland, MD). hCG was administered when a transvaginal ultrasound revealed the largest follicle had a mean diameter of ≥18 mm. The insemination was performed in a sterile fashion, using a flexible plastic catheter with the patient in the dorsal lithotomy position. The patient remained supine for at least ten minutes after the end of the insemination.

Serum β-hCG levels were analyzed 15 to 17 days after IUI to determine pregnancy status. Blood samples were assayed on an Immulite 2500 (Diagnostic Products Corporation, Los Angeles, CA) for a quantitative measurement of β-hCG. The Immulite uses a solid-phase two-site chemiluminescent immunometric assay with a sensitivity of 1 mIU/mL and a calibrated range to 5000 mIU/mL. Intra- and inter-assay coefficients of variation were each less than 7%. Most normal singleton pregnancies have levels in the range of 50 to 100 mIU/mL at this gestation. However, a level higher than five mIU/mL was considered positive for pregnancy.

### Statistical analysis

All statistical analyses were performed using the Statistical Package for the Social Sciences 11.0 (SPSS, Inc., Chicago, IL). Continuous variables were evaluated for normal distribution using the Kolmogorov-Smirnov test. Any variables that were not normally distributed were logarithmically transformed to obtain normality. Results are reported as mean value ± standard deviation (SD). Categorical variables were evaluated with likelihood ratios. Likelihood ratios were calculated as:

LR+ = / sensitivity / 1-specificity

which is equivalent to;

LR+ = / Pr (T+/D+) / Pr (T+/D-)

or “the probability of a person who falls into a grouping of the semen analysis having a pregnancy divided by the probability of a person who does not fall into the semen grouping having a pregnancy.” Here “T+” or “T−” denotes that the classification into the semen analysis grouping is positive or negative, respectively. Likewise, “D+” or “D−” denotes that the pregnancy is present or absent, respectively. T-tests were used to compare for continuous variables. Levine’s test for equality of variances was used to determine which p value to accept. Significance was taken as a p≤0.05.

### Ethical approval

The university’s Human Subjects Research Ethics Committee approved this study (IRB number 95940). The authors have no conflict of interest.

## Results

Baseline data of the cohort are provided in Table 1. An initial comparison without controlling for other semen analysis results was made to determine any single abnormal factor that gave lower pregnancy rates. Those with and without a pregnancy were classified based on volume <1.5 mL or not, concentration <15 mil/mL or not, <32% forward motility or not, and <39 million sperm in the specimen. The results are presented in Table 2. Data are presented as mean values and SDs in the pregnant and not pregnant groups. The p-values for the likelihood ratio (one-sided, because it was hypothesized that abnormal results would have lower pregnancy values) are also presented comparing pregnancy rates in the groups that were normal or abnormal for the given parameter. As expected, the parameters were significantly different when comparing those grouped based on a parameter being abnormal or not. Among the parameters, only total sperm in specimens with <39 million gave lower pregnancy rates. 

Next, semen analysis results were categorized based on the presence of one or more abnormal parameters, and precisely what parameters were abnormal. This gave the ability to control for confounding effects. At this stage, comparison was performed using volume (less than or greater than 1.5 mL), concentration (less than or greater than 15 mil/mL), and forward motility (less than or greater than 32%). For this comparison, it was elected to exclude total sperm count because this value is not traditionally presented in a standard semen analysis reports. The results are presented in Table 3. Pregnancy rates are shown comparing all parameters in the normal group. As can be noted, none of the parameters or combination of these parameters predicted lower pregnancy rates when compared with normal specimens. Although two of the groups comprised few patients, given the trends in the total results, it is unlikely that the small numbers were the cause of lack of significance. The semen parameters for these seven groupings are presented in Table 4 for patients with and without pregnancies.

We made a comparison using total sperm count of less than or at least 39 million as well as volume and motility as predictors of pregnancy when compared with the normal group for all 3 because total sperm count in the specimen was the only factor that seemed to be associated with pregnancy rates. These results are shown in Table 5. There are fewer comparisons performed than in Table 3 because we did not repeat any comparisons already presented. Consideration of sperm concentration was not performed. It should be noted than only the groups with total counts less than 39 million, motility less than 32%, and volume less than 1.5 mL had a lower pregnancy rate. Even the group with low total count and motility but normal volume was not associated with pregnancy outcome, even though this group’s results were equivalent to a low TMSC by the 2010 WHO parameters. Table 6 presents the semen analysis parameters from this group.

## Discussion

Semen analysis has been the subject of debate for many years. It is unclear whether applying parameters found in a fertile population to an infertile population is valid (13,14,32). However, to this day, semen analysis remains the primary objective measure of male factor infertility. For this reason, this study was performed to determine the relationship between abnormal semen parameters and pregnancy rates in couples undergoing IUI. Our study is the first of its kind, making it unique in nature, using the 2010 WHO parameters.

The results demonstrate that if a single parameter is abnormal among those traditionally used to evaluate semen analysis, then pregnancy rates are unaffected with the exclusion of total sperm count less than 39 million in the specimen (Table 2). The data would be stronger if abnormal Kruger-Tyberg strict morphology data were available. However, because these patients are treated with IVF and ICSI at the center, conclusions cannot be drawn related to morphology. It remains important to note that the total quantity of sperm in the specimen affects pregnancy rates while other factors do not. Furthermore, pregnancy rates remain acceptable at 16% (p=0.4, Table 5).

Studies have found that TMSC was among the most important predictive factors of successful pregnancy rates (12,15,16,17,18,19,20,21,22). In Table 5, an evaluation of the parameters used to calculate TMSC is presented. When the total count and motility were low (which equates with a measure of low TMSC), pregnancy rates were 18% and remained unaffected when compared with the normal group. This likely occurred because if measured then the TMSC would be abnormal once the level was below 12.48 million sperm. Most of the studies listed above only found decreased pregnancy rates when the TMSC was less than 10 million (17,18,19,21) or 5 million (15,16,20,22), which is well below the normal parameters quoted in the 2010 WHO guidelines. 

The value of TMSC in IUI nevertheless remains debated. Khalil et al. (16) in a retrospective study found that a TMSC of 5 million or higher was associated with higher pregnancy rates. In a descriptive retrospective cohort study by Kleppe et al. (23) based on 895 cycles in 273 couples, the cumulative pregnancy rates increased from 17.3% as opposed to 25.5% with TMSC less than 1 million and greater than 1 million. Clearly, a 17% pregnancy rate with a TMSC under a million remains an acceptable percentage. Pasqualotto et al. (24) concluded that the live birth rate increased with increased TMSC. However, they commented on the fact that success from IUI was mainly related to the percentage of motile sperm (24). 

Typically, moderate male factor infertility is considered present when more than a single factor is abnormal (13,25,26) and therefore, one would expect to see decreased pregnancy rates in this situation. However, our results show similar pregnancy rates when one, two, and in most cases three parameters were abnormal. The exception occurred if the total count was less than 39 million sperm, the volume was less than 1.5 mL, and the forward motility was less than 32%, in which case pregnancy rates decreased significantly, although remaining acceptable. Therefore, a couple with mild-to-moderate male factor infertility should be offered IUI as we would expect similar pregnancy rates as quoted in the literature of 13-20% (6,23,27). Pregnancy rates in this study ranged from 11-25%, excluding those that had a total sperm count less than 39 million, plus volume and forward motility also being abnormal. These pregnancy rates are evidently acceptable. Therefore, these rates play an important role in counseling couples when they present for assistive reproductive technology treatments. 

There is significant debate as to whether sperm concentration affects pregnancy rates. The literature suggests a direct relationship with the number of spermatozoa in the specimen and pregnancy rates (20,28). However, the results of the present study demonstrate that when sperm concentration is the single abnormal parameter, pregnancy rates (17%) are excellent. Dorjpurev et al. (19) found slightly lower pregnancy rates per cycle when comparing sperm concentration of <20 × 106/mL (4.1%) vs ³20 × 106/mL (7.3%). In a prospective study, Haim et al. (29) showed that there was no significant difference in pregnancy rates with increasing sperm concentration. Pregnancy rates were 7.5% with concentration <10 × 106/mL, whereas they were 10.9% when concentrations were >40 × 106/mL (29). Therefore, sperm concentration does not impact pregnancy rates significantly and, rather, TMSC is more predictive of successful IUI cycle. 

A parameter that was not considered and poses a limitation to this study was sperm morphology. The majority of studies have consistently shown that sperm morphology is one of the best predictors of IVF and IUI outcomes (6). Coetzee et al. (30) demonstrated through a literature review that overall fertilization rates were 59.3% when morphology was <4% and 77.6% when >4% and pregnancy rates were 15.2% and 26.0%, respectively. A literature review by Van Waart et al. (6) concluded that the tendency to become pregnant when sperm morphology was ≤4% was significantly decreased, and this was further supported by a review conducted by van der Merwe et al. (31) who concluded that morphology was the best predictor of sub-fertility and that a cut-off of <5% should be used. However, sperm morphology is not traditionally calculated on the day of IUI because to do so would require killing a significant part of the specimen. It should also be noted that this population had a strict morphology on a recent semen analysis ≥4%, which places them in the WHO normal range.

Another weakness of the study was the small number of subjects in particular groupings of semen parameters. Although these small numbers make it hard to conclude about the grouping individually, consistencies in the data as a whole are visible, notably the lack of differences. Nevertheless, confirmation of the results based on an even larger study would be helpful.

Data were purposefully not presented on female parameters or the stimulation protocol used. Slightly less than 2% used natural cycle IUI. The remaining patients used clomiphene, letrozole or gonadotropins. These data were not provided because it most closely resembles patient counseling on the day of IUI, based on the sperm. The physician cannot interpret the interplay of maternal age, body mass index, years of infertility and stimulation protocol, combined with semen analysis parameters. The physician instead states the sperm parameters and as such whether pregnancy rates are normal or diminished. This study permits an evidence-based interpretation of these parameters on the day of IUI, for the first time. It should be noted that one of the factors that affect pregnancy rates obtained with IUI cycles include stimulation medications. Pregnancy rates are often lower with oral drugs and higher with gonadotropins. In theory, the non-inclusion of these parameters represent a weakness of this study. However, by maintaining the premise that physicians counsel patients based only on semen parameters on the day of IUI, stimulation medications are not taken into consideration and as such were not included in the analysis.

Concurrently, clinical pregnancy rates are not presented because they are affected by factors that do not necessarily affect the pregnancy rate, i.e., sperm DNA fragmentation, history of recurrent pregnancy loss, uterine anomalies, and endometrial quality, among other factors (33,34,35). Lastly, this is not an examination of multiple pregnancies in IUI cycles, just the likelihood of pregnancy, based on semen parameters on IUI day. To evaluate the effect of semen parameters on multiple pregnancy rates with IUI is an interesting study; however, do to this study would require significant space and is worthy of its own paper. 

One question that arises is whether biochemical pregnancy or clinical pregnancy should be used to measure semen parameter-related success. In our study, pregnancy rates were determined using serum β-hCG results, rather than with evidence of clinical pregnancy or live birth. However, it can be countered that semen capability is best measured in fertilization and biochemical pregnancy, whereas clinical pregnancy or live birth depends more on uterine environment, maternal age, and embryo developmental capacity. All these factors are sperm independent.

In conclusion, IUI remains an effective treatment when faced with a couple with male factor infertility. In all situations, pregnancy rates were at least 11% per cycle and therefore, certain abnormal semen analysis parameters should not be used to discourage IUI. Total sperm in the specimen and not the concentration of sperm per milliliter was the essential factor for predicting pregnancy. Therefore, a reorganization of the semen analysis report should be made emphasizing the total amount of sperm present and de-emphasizing the concentration of sperm. 

## Figures and Tables

**Table 1:**
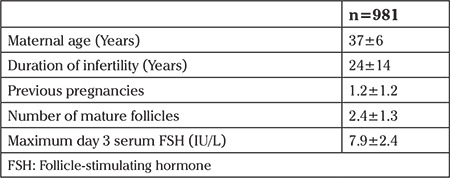
The baseline characteristics of subjects

**Table 2:**
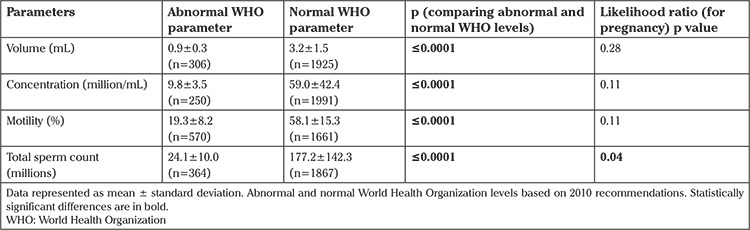
Comparisons of pregnancy rates and parameters in the groups abnormal for any of the listed criteria according to the 2010 World Health Organization semen analysis criteria without controlling for other semen analysis parameters

**Table 3:**
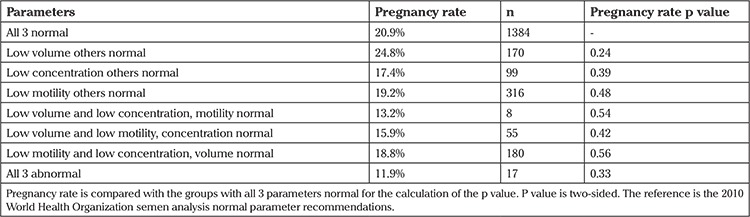
A comparison of data and pregnancy rates when one or more of the traditionally reported semen parameters are abnormal

**Table 4:**
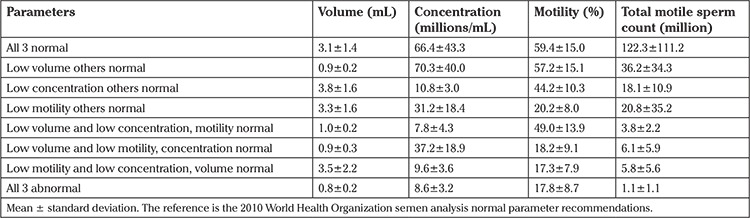
Preprocessing semen characteristics on the day of intrauterine insemination for the 7 different groupings

**Table 5:**
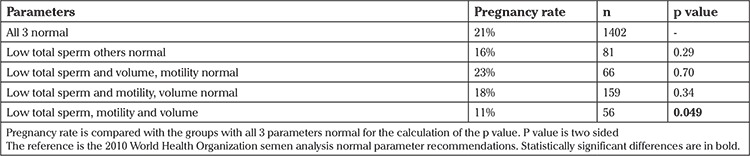
Comparison of pregnancy rates based on total sperm count in specimen, volume and motility

**Table 6:**
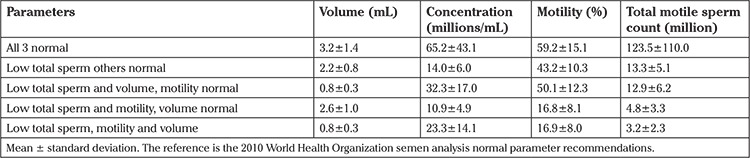
Preprocessing semen characteristics on the day of intra uterine inseminations for the different groups based on total sperm count in the specimen
